# Supplementary material for: Microbiome of Citrullus colocynthis (L.) Schrad. Reveals a Potential Association with Non-Photosynthetic Cyanobacteria
Source: Microorganisms. 2022 Oct 21;10(10):2083. doi: 10.3390/microorganisms10102083 (PMC9607294; doi:10.3390/microorganisms10102083)
Supplement: Supplementary file 1 [file microorganisms-10-02083-s001.zip › Supplementary Figures S1 to S3.pdf]

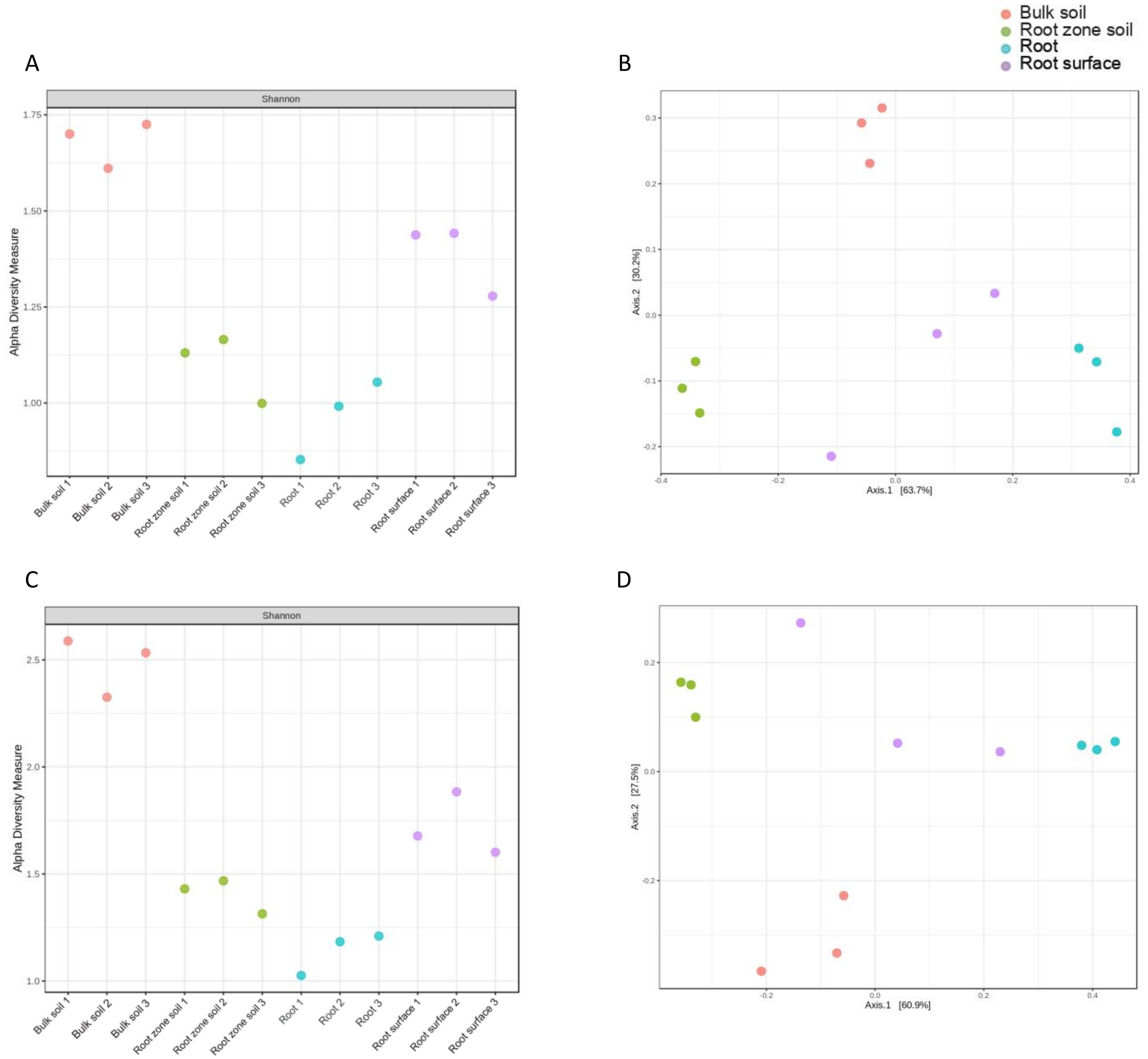

Supplementary Figure S1. A) Shannon Alpha diversity and B) principal coordinates analysis (PCoA) for beta-diversity (B) at the species level of root and soil samples from three different *C. colocynthis* plants. Alpha diversity metrics (p-value: 3.8567e-05; [ANOVA] F-value: 39.441); beta diversity metrics ([PERMANOVA] F-value: 19.027; R-squared: 0.87708; p-value: 0.001). C) Alpha diversity and D) principal coordinates analysis (PCoA) for beta-diversity (B) at the class level of root and soil samples from three different *C. colocynthis* plants. Alpha diversity metrics (p-value: 4.1118e-06; [ANOVA] F-value: 71.234); beta diversity metrics ([PERMANOVA] F-value: 14.348; R-squared: 0.84327; p-value: 0.001).

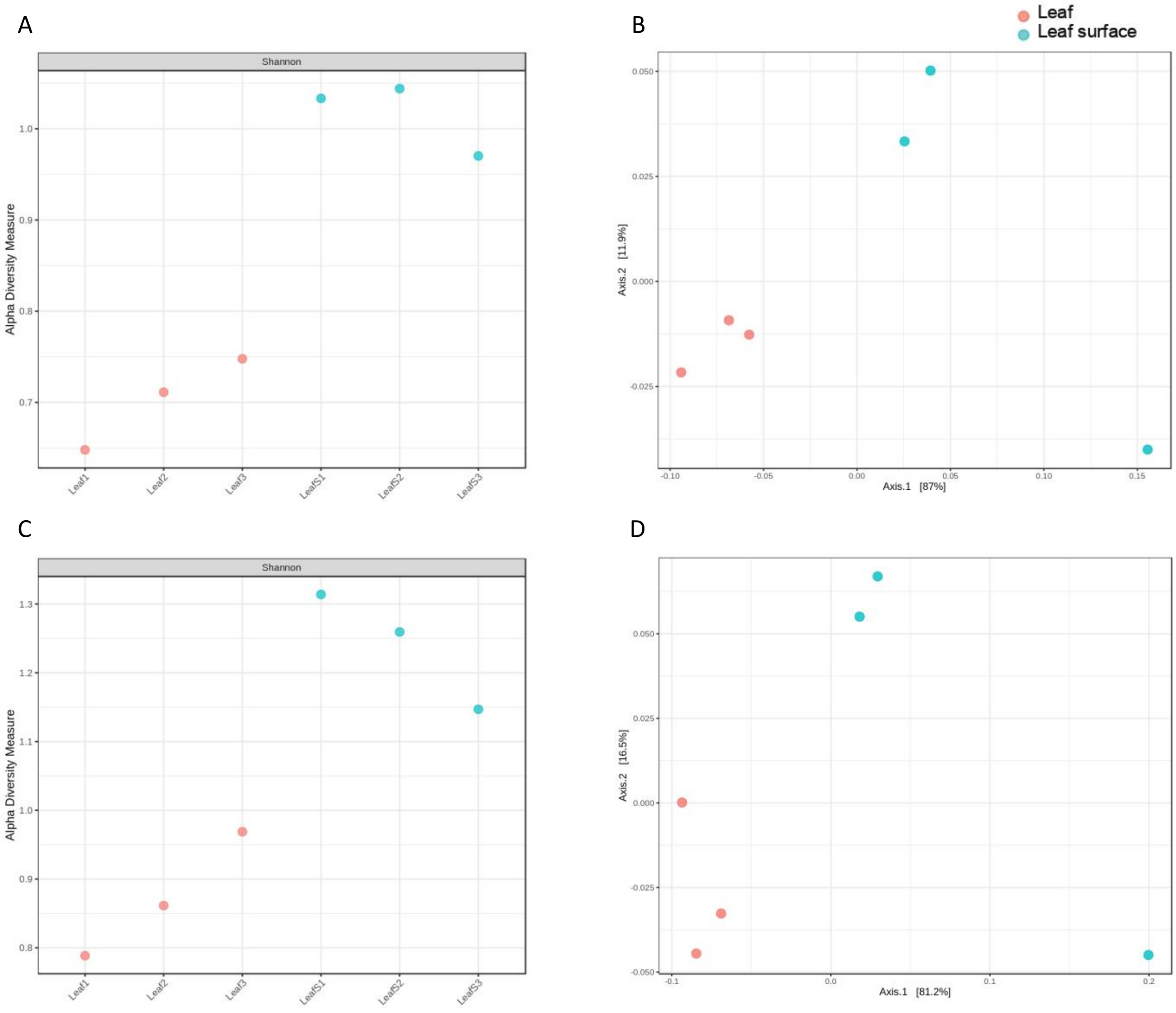

Supplementary Figure S2. A) Shannon Alpha diversity and B) principal coordinates analysis (PCoA) for beta-diversity (B) at the phylum level of leaf and leaf surface samples from three different *C. colocynthis* plants. Alpha diversity metrics (p-value: 0.0013542; [T-test] statistic: -8.4401); beta diversity metrics ([PERMANOVA] F-value: 8.3481; R-squared: 0.67606; p-value: 0.1). C) Alpha diversity and D) principal coordinates analysis (PCoA) for beta-diversity (B) at the class level of leaf and leaf surface samples from three different *C. colocynthis* plants. Alpha diversity metrics (p-value: 0.0070091; [T-test] statistic: -5.1096); beta diversity metrics ([PERMANOVA] F-value: 5.6967; R-squared: 0.58749; p-value: 0.1).

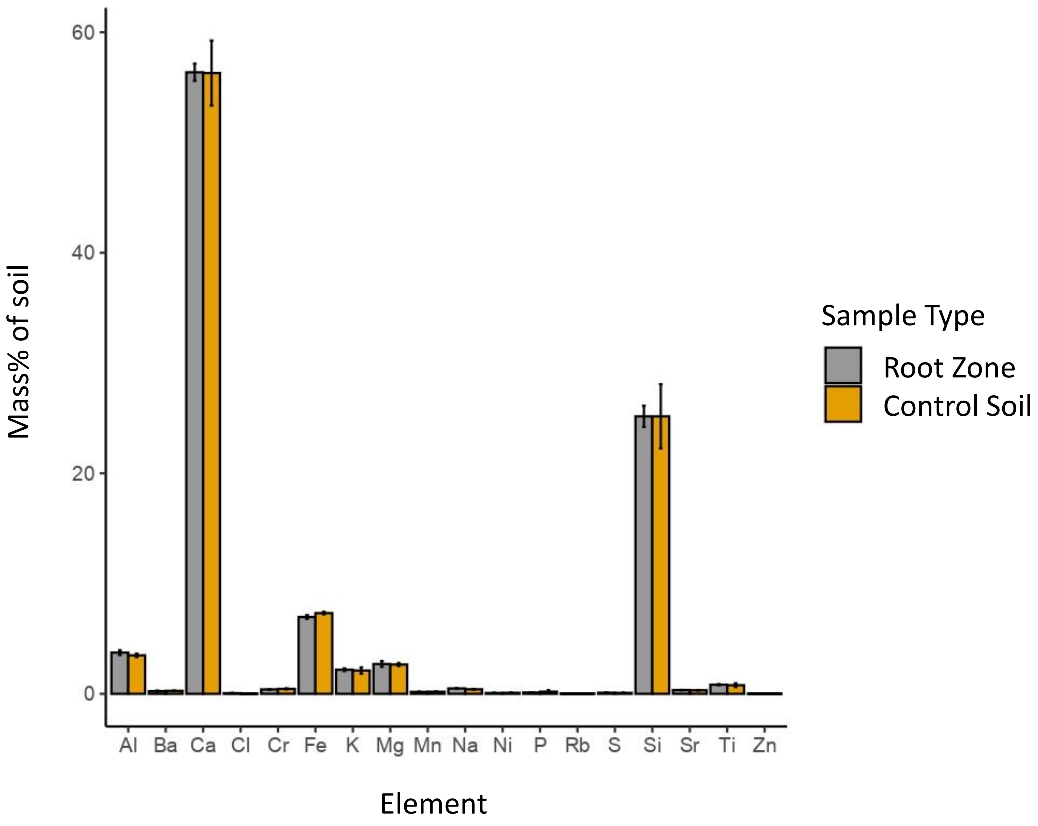

Supplementary Figure S3. Elemental composition of soil samples collected from the root zone of *C. colocynthis* and control (bulk) soil, expressed in mass%.
